# Neural tube closure depends on expression of Grainyhead-like 3 in multiple tissues

**DOI:** 10.1016/j.ydbio.2018.01.016

**Published:** 2018-03-15

**Authors:** Sandra C.P. De Castro, Caroline S. Hirst, Dawn Savery, Ana Rolo, Heiko Lickert, Bogi Andersen, Andrew J. Copp, Nicholas D.E. Greene

**Affiliations:** a Developmental Biology&Cancer Programme, UCL Great Ormond Street Institute of Child Health, University College London, London, WC1N 1EH, UK; b HelmholtzZentrum München, Deutsches Forschungszentrum für Gesundheit und Umwelt (GmbH) Business Campus Garching, Parkring 11, 85748 Garching near Munich, Germany; c Department of Biological Chemistry, School of Medicine, University of California Irvine, Irvine, California, USA; d Department of Medicine, School of Medicine, University of California Irvine, Irvine, California, USA

**Keywords:** Neural tube defects, grainyhead, spina bifida, curly tail, mouse embryo

## Abstract

Failure of neural tube closure leads to neural tube defects (NTDs), common congenital abnormalities in humans. Among the genes whose loss of function causes NTDs in mice, Grainyhead-like3 (*Grhl3*) is essential for spinal neural tube closure, with null mutants exhibiting fully penetrant spina bifida. During spinal neurulation *Grhl3* is initially expressed in the surface (non-neural) ectoderm, subsequently in the neuroepithelial component of the neural folds and at the node-streak border, and finally in the hindgut endoderm. Here, we show that endoderm-specific knockout of *Grhl3* causes late-arising spinal NTDs, preceded by increased ventral curvature of the caudal region which was shown previously to suppress closure of the spinal neural folds. This finding supports the hypothesis that diminished *Grhl3* expression in the hindgut is the cause of spinal NTDs in the *curly tail,* carrying a hypomorphic *Grhl3* allele. Complete loss of Grhl3 function produces a more severe phenotype in which closure fails earlier in neurulation, before the stage of onset of expression in the hindgut of wild-type embryos. This implicates additional tissues and NTD mechanisms in *Grhl3* null embryos. Conditional knockout of *Grhl3* in the neural plate and node-streak border has minimal effect on closure, suggesting that abnormal function of surface ectoderm, where Grhl3 transcripts are first detected, is primarily responsible for early failure of spinal neurulation in *Grhl3* null embryos.

## Highlights

• Conditional knockout of *Grhl3* in the hindgut causes spinal NTDs owing to incomplete closure of the posterior neuropore late in spinal neurulation.
• On the other hand, closure fails early in spinal neurulation in *Grhl3* null embryos, prior to the normal stage of hindgut expression.
• Stage-dependent analysis of *Grhl3* expression implicates the non-neural ectoderm in the early failure of closure.
• Grhl3 is also expressed in neural plate and neuromesodermal precursors, but knock-out in these tissues does not cause NTDs.

## 1 Introduction

In higher vertebrates neural tube closure is achieved through coordinated shaping and bending of the neural plate to form bilateral neural folds which adhere in the midline (Greene and Copp, 2014, Nikolopoulou et al., 2017). Closure propagates between initial sites of adhesion to seal the intervening open regions, termed neuropores. Failure to complete closure results in severe birth defects, termed neural tube defects (NTDs), which include spina bifida and anencephaly (Copp et al., 2013). The closure process is not solely dependent on intrinsic properties of the neuroepithelium (Pai et al., 2012, Ray and Niswander, 2012). The leading edges of the neural folds at the closure site comprise neuroepithelium with overlying surface (non-neural) ectoderm and intervening extracellular matrix. Notably, initial contact appears to be mediated by surface ectoderm cells at the boundary with the neuroepithelium at both cranial and spinal levels (Ray and Niswander, 2016, Rolo et al., 2016).

Among the genes required for neural tube closure, members of the *grainyhead-like* family of transcription factors (Gustavsson et al., 2008, Ting et al., 2003a) appear essential at both cranial and spinal levels of the body axis. For example, mice lacking expression of *Grhl3* (previously *Get1* or *Som*) exhibit fully penetrant spina bifida, with a low frequency of exencephaly, the developmental forerunner of anencephaly (Ting et al., 2003a, Ting et al., 2003b; Yu et al., 2006).

At later stages of development and post-natally, Grhl3 has key functions in epithelia, being required for differentiation of the epidermis during late-fetal development. Knockouts display defective barrier formation, as well as impaired wound healing (Gordon et al., 2014, Hopkin et al., 2012, Ting et al., 2005; Yu et al., 2006). The function of *Grhl3* in epidermal differentiation parallels the requirement for *Drosophila grainyhead* (*grh*) in regulating cuticle development (Uv et al., 1997). Although attention has focussed on the role of *Grhl3* in surface ectoderm and skin, expression is also present in epithelia of the gastrointestinal tract, bladder and lung (Kudryavtseva et al., 2003). *Cdh1* (encoding E-cadherin), a marker of surface ectoderm and epithelia, is a direct target of *Grhl3* in mouse mammary gland cells (Alotaibi et al., 2015).

At neurulation stages, *Grhl3* is expressed from the earliest stages in the surface ectoderm, in keeping with its later function in epidermal differentiation. However, it is also expressed in other sites including transiently in the spinal neuroepithelium and in the hindgut endoderm (Gustavsson et al., 2007, Gustavsson et al., 2008, Ting et al., 2003a, Ting et al., 2003b). The identification of Grhl3 expression in several tissues during neurulation raises questions over which expression site(s) is essential for neural tube closure. For example, it has been proposed that Grhl3 contributes to delineation of the border between surface ectoderm and the neuroepithelium (Kimura-Yoshida et al., 2015).

A requirement for Grhl3 function in the hindgut endoderm was suggested by analysis of the *curly tail* (*ct*) mouse strain, which carries a hypomorphic allele of *Grhl3* and exhibits partially penetrant spinal NTDs (Gustavsson et al., 2007, Van Straaten and Copp, 2001). Neural tube closure is apparently unaffected in approximately 50% of homozygous *ct/ct* embryos, while affected embryos can be recognised by the presence of an enlarged posterior neuropore (PNP) at embryonic day 10.5 (E10.5; Copp, 1985). This is indicative of subsequent failure or delay of closure of the spinal neural tube, which lead to spina bifida (approximately 15% of embryos) or a tail flexion defect (approximately 40%), respectively. Failure of PNP closure in *ct/ct* embryos correlates with reduced cellular proliferation rate in the hindgut endoderm and notochord (Copp et al., 1988a). In combination with ‘normal’ proliferation in the neuroepithelium, this causes a growth imbalance between dorsal and ventral tissues leading to increased ventral curvature of the caudal region of the embryo. Increased curvature mechanically opposes closure, as demonstrated in both mouse and chick embryos (Van Straaten et al., 1993); indeed, splinting of the caudal region allows PNP closure to progress normally in cultured *ct/ct* embryos (Brook et al., 1991). Moreover, normalisation of the growth imbalance, either through suppression (Copp et al., 1988b) or stimulation of proliferation (Cogram et al., 2004, Leung et al., 2013), prevents spinal NTDs in the *ct* mutant.

*Grhl3* expression is diminished in *ct/ct* embryos and their spinal NTDs can be prevented by BAC transgene-mediated expression of *Grhl3* (Gustavsson et al., 2007). In transgenic embryos, *Grhl3* expression was increased in each of the endogenous sites of expression, therefore leaving open the question of whether *Grhl3* expression is required in tissues other than the hindgut for neural tube closure. Moreover, the frequency of NTDs in the *ct/ct* strain is strongly influenced by genetic modifiers, raising the possibility that one of these is responsible for the hindgut phenotype (Letts et al., 1995, Neumann et al., 1994, Van Straaten and Copp, 2001, de Castro et al., 2012). These considerations led us to investigate the tissue-specific requirements for *Grhl3* function in neural tube closure, in order to ask whether expression is needed in more than one tissue, possibly at different developmental stages.

## 2 Materials and Methods

### 2.1 Mice

A conditional (floxed) allele of *Grhl3* (designated *Grhl3*^*f/+*^) has been described (Yu et al., 2006). These mice were crossed to β-actin-*Cre* mice to generate heterozygous null, *Grhl3*^+/-^, mice used in subsequent experimental matings. Tissue-specific cre-driver lines were *Sox17-2A-iCre* (Engert et al., 2009), *Nkx1-2-Cre* (Albors et al. http://dx.doi.org/10.1101/045872) and *Grhl3-Cre* (Camerer et al., 2010). *Cre* lines were validated by crosses to mice carrying the *Gt(ROSA)26Sor*^*tm1(EYFP)C*^*°*^*s*^ reporter allele (Srinivas et al., 2001). Mice were genotyped by PCR of genomic DNA, as described (Camerer et al., 2010, Gustavsson et al., 2007; Yu et al., 2006).

Animal studies were carried out under regulations of the Animals (Scientific Procedures) Act 1986 of the UK Government, and in accordance with guidance issued by the Medical Research Council, UK in *Responsibility in the Use of Animals for Medical Research* (July 1993). Litters were generated by timed matings in which mice were paired overnight and the day of finding a copulation plug was designated embryonic day 0.5 (E0.5). YFP expression in whole mount embryos was visualised by direct fluorescence. Embryos for *in situ* hybridisation were rinsed in phosphate buffered saline (PBS) and fixed in 4% paraformaldehyde (PFA) in PBS at 4 °C overnight.

### 2.2 Morphological measurements

The PNP length of embryos at E8.5 to E10.5 was measured using an eye-piece graticule, from the tip of the tail bud to the rostral limit of the open neural folds. For measurements of ventral curvature, the caudal region of E9.5-10.5 embryos was isolated from the body, photographed and curvature of the caudal region measured as described (Brook et al., 1991, Brouns et al., 2011).

**Whole mount** ***in situ*** **hybridisation** was performed as previously reported (de Castro et al., 2012, Gustavsson et al., 2007), following which embryos were embedded in albumin-gelatine and 40 µm sections obtained on a vibratome. Images were processed using Photoshop (Version 6.0) for cropping and figures were prepared using Adobe Illustrator software.

### 2.3 Quantitative real time RT-PCR (qRT-PCR)

RNA was isolated from the caudal region of E8.5 (10-14ss, cut at somite 10), E9.5 (15-16ss, cut at somite 12) and E10.5 (26-31ss, cut at somite 14) embryos. Total RNA was isolated using TRIzol Reagent (Gibco) followed by DNase treatment (DNA-free, Ambion). Primers for qRT-PCR were designed to give a product of 150–250 bp (sequences available on request) and optimised for 60°C annealing temperature. RNA extraction, cDNA synthesis and quantitative RT-PCR were performed using glyceraldehyde-3-phosphate dehydrogenase (*Gapdh*) for normalisation, as previously (Gustavsson et al., 2007, Brouns et al., 2011). qRT-PCR used iTAQ Universal SYBR Green Supermix assay (Bio-Rad) on a CFX96 system (Bio-Rad) with analysis using Bio-Rad CFX Manager software. Individual experiments were combined and analysed using Sigma Stat software (ANOVA or t-test).

## 3 Results

### 3.1 Excess body curvature in *Grhl3* null embryos

**Fig. 1:**
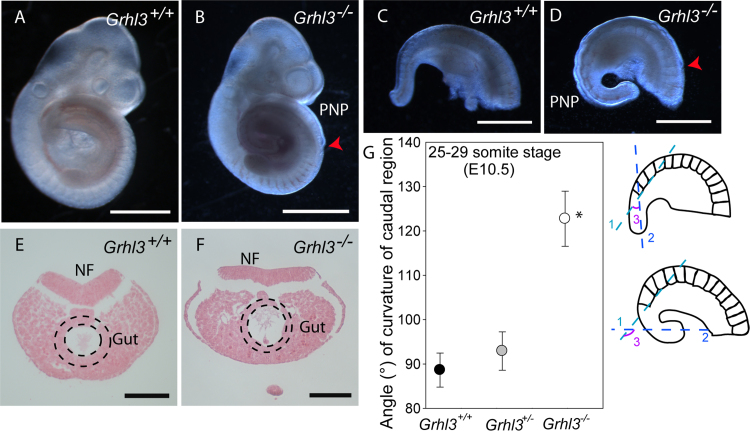
**Embryonic curvature of the caudal region is enhanced in*****Grhl3*****null embryos.** (A-D) Spinal neurulation is complete in wild-type embryos at E10.5 (A, C), whereas the PNP of *Grhl3*^*-/-*^ embryos remains persistently open (B, D; red arrowhead indicates rostral limit of PNP). The caudal region of *Grhl3*^*-/-*^ embryos appears excessively curved (compare C, D). (E, F) Transverse sections in E-F through the open PNP region of embryos at E10.5; the PNP is open in both wild-type and *Grhl3*^*-/-*^ embryos. Hindgut is indicated by dashed lines. (G) At E10.5 (stage-matched at 25–29 somites), the angle of ventral curvature, ‘3’, was determined between line 1, drawn tangential to the ventral edge of the penultimate somite and line 2, drawn along the midline of the tail bud, parallel to and equidistant from the ventral and dorsal surfaces. The mean (±SEM) angle of curvature of the caudal region of *Grhl3*^*-/-*^ embryos (n= 4) is significantly greater than among wild-type (n = 8) and heterozygous (n = 12) littermates (* p<0.001, One Way ANOVA). Mean±SEM is shown for each genotype. Scale bar = 1 mm (A-B), 0.5 mm (C-D) or 0.1 mm (E-F).

Genetic mapping and transgenic rescue provide evidence that the major genetic cause of NTDs in *ct/ct* embryos is a hypomorphic allele of *Grhl3* (here denoted *Grhl3*^*ct*^). In this model, the causative cellular mechanism is thought to be diminished proliferation in the hindgut and consequent excess curvature of the caudal region that opposes closure (see above; Van Straaten and Copp, 2001). However, it remained formally possible that reduced proliferation in the hindgut was a result of modifier gene action with NTDs resulting from summation with a deleterious effect of insufficient *Grhl3* expression in another tissue. We investigated this question in embryos carrying combinations of null or ‘floxed’ alleles of *Grhl3* (Yu et al., 2006). We first examined litters from intercross of *Grhl3*^*+/-*^ mice. At late E10.5, the caudal region of *Grhl3*^*-/-*^ embryos showed obvious excess curvature (Fig. 1A-D) and the posterior neuropore (PNP) remained extensively open (Fig. 1B, D), whereas closure is complete in all wild-type embryos at this stage. Earlier on E10.5, when PNP closure is still incomplete in wild-type and *Grhl3*^*-/-*^ embryos (Fig. 1E, F), the latter already showed a significantly increased ventral curvature of the caudal region (Fig. 1G). These observations are consistent with the hypothesis that diminished *Grhl3* expression can affect axial curvature, as observed in affected *ct* embryos at the same stage (Brook et al., 1991). Hence, increased curvature in *ct/ct* embryos is unlikely to be due solely to genetic modifiers.

### 3.2 Loss of *Grhl3* expression in the gut endoderm is sufficient to cause spinal NTDs

**Fig. 2:**
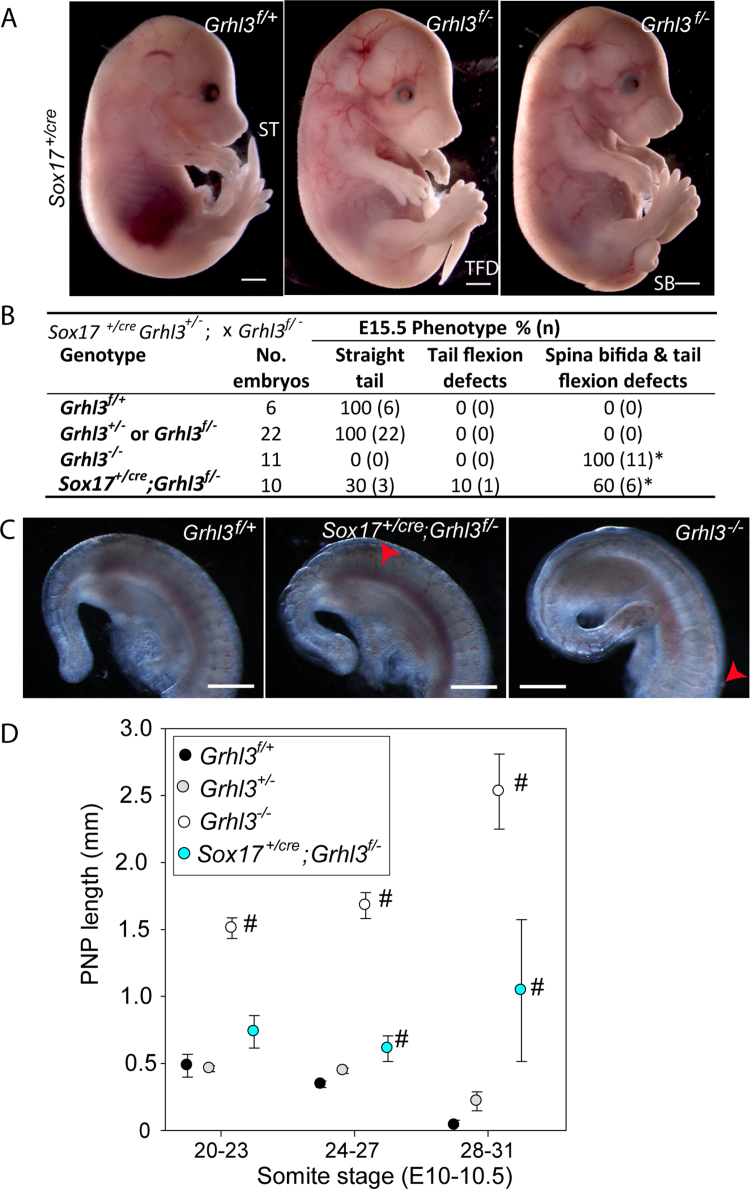
**Loss of*****Grhl3*****expression in gut endoderm causes NTDs.** (A, B) Among litters analysed at E15.5, conditional knockout of *Grhl3* in the gut endoderm, using *Sox17*^*cre*^ causes spina bifida (SB; example shown also has a tightly curled tail) and/or tail flexion defects (TFD) whereas wild-type and heterozygotes are unaffected (ST; straight tail; scale bars represent 1 mm). The proportion of phenotypes differs significantly with genotype (* different from wild-type and *Grhl3* heterozygous genotypes, p<0.001; Fisher Exact). (C) At E11.5, both *Sox17*^*+/cre*^;*Grhl3*^*f/-*^ and *Grhl3*^*-/-*^ embryos have persistent open, extremely large PNPs (rostral limit indicated by red arrowhead), whereas the PNP is closed in wild-type embryos (scale bars = 0.5 mm). (D) At E10.5, the PNP of wildtype and heterozygous embryos shortens as closure progresses to completion. PNP length of *Grhl3* null (*Grhl3*^*-/-*^) is enlarged at all somite stages, and and gut-conditional null (*Sox17*^*+/cre*^;*Grhl3*^*f/-*^) embryos is enlarged from 24 somites onwards compared with wild-type (*Grhl3*^*f/+*^) and heterozygous embryos. # indicates significant difference compared with all other genotypes (p<0.001; ANOVA); n = 15 *Grhl3*^*f/+*^, 57 *Grhl3*^*+/-*^, 28 *Grhl3*^*-/-*^, 31 *Sox17*^*cre/+*^*; Grhl3*^*f/-*^ (5–28 embryos per group at each somite stage).

Next, we directly tested the requirement for *Grhl3* expression in the hindgut by conditional deletion of *Grhl3* in the endoderm. A ‘floxed’ allele of *Grhl3*, designated *Grhl3*^*f*^ (Yu et al., 2006) was recombined by *Sox17-2A-iCre*, which is active in the hindgut from E8.5 but not in other *Grhl3* expression sites (Engert et al., 2009). Litters from matings of *Sox17*^*Cre/+*^; *Grhl3*^*+/-*^ with *Grhl3*^*f/-*^ mice (dams included either parental genotype) were analysed at E11.5-15.5 (Fig. 2A-C). NTDs were not observed among Cre-negative *Grhl3*^*+/-*^ or *Grhl3*^*f/-*^ embryos (both genotypes are effectively heterozygous), whereas spina bifida occurred in 60% (6/10 embryos) and tail flexion defects in 70% (7/10) of *Sox17*^*Cre/+*^; *Grhl3*^*f/-*^ embryos. We conclude that loss of *Grhl3* expression in the gut endoderm is sufficient to cause NTDs.

**Fig. 3:**
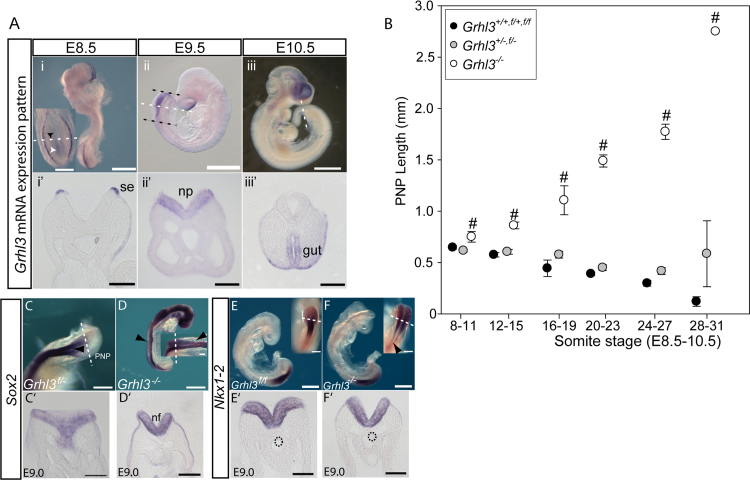
**Grhl3 is required for spinal neurulation prior to expression in neural plate and/or hindgut.** (A) *Grhl3* exhibits a dynamic expression pattern during neurulation. Expression is evident in the surface ectoderm on the outside of the spinal neural folds, and at E8.5 in the caudal lateral epiblast (white arrowhead) as well as some cells in the neuroepithelium (black arrowhead). Expression is detected in the neuroepithelium (np) at E9.5 and hindgut at E10.5. Dashed white lines in i-iii indicate level of sections in i’-iii’; scale bars represent 0.5 mm in i-iii and 0.1 mm in i’-iii’. PNP length delineated by dashed black lines in Aii. (B) Analysis at E8.5-10.5 shows that the mean PNP length of *Grhl3* null embryos (n = 78) is significantly enlarged from the 8–11 somite stage onwards compared with *Grhl3* heterozygous (*Grhl3*^*+/-*^ and *Grhl3*^*f/-*^; n = 152) and wild-type (n = 100) littermates (# significantly different compared with other genotypes; p<0.001, ANOVA; n = 10–47 embryos at each somite interval except 2–5 embryos at 28–31 somite stage). (C-F) Expression of *Sox2* (C-D) and *Nkx1-2* (E-F) does not differ in E9.0 *Grhl3*^*-/-*^ embryos (D, F) from other genotypes (C, E). Transverse sections (C’-F’; at level of white dashed lines in C-F) show expression of both genes in the open neural folds (nf). Hindgut is outlined by black dashed lines in E-F. Scale bars represent 0.5 mm in D-F; and 0.1 mm in C and C’-F’.

In order to confirm that gut-conditional NTDs result from failure of PNP closure, rather than later re-opening of a closed neural tube, additional litters were collected at E10-10.5. Measurements were made of PNP length (Fig. 2D), an enlarged PNP being indicative of ensuing delay or failure of closure (Copp, 1985). The mean PNP length of *Sox17*^*Cre/+*^*; Grhl3*^*f/-*^ embryos became abnormally enlarged from the 24–27 somite stage (Fig. 2D; Fig. S2), consistent with failure of the normal onset of *Grhl3* expression in the hindgut endoderm from this stage (Fig. 3A).

### 3.3 *Grhl3* is required in multiple tissues during neural tube closure

All *Grhl3*^*-/-*^ fetuses generated in the experimental intercross of *Sox17*^*Cre/+*^*; Grhl3*^*f/-*^ with *Grhl3*^*f/-*^ developed spina bifida (Fig. 2B) and the extent of the open spinal lesion appeared greater than in hindgut-conditional mutants (examples shown in Fig. 2C). Notably, PNP length was already significantly enlarged in *Grhl3*^*-/-*^ embryos at 20–23 somites, in contrast to hindgut-conditional embryos whose PNP length had not yet become different from wild-type and heterozygous embryos (Fig. 2D). Hence, failure of PNP closure occurs at an earlier stage in the constitutive null embryos than in hindgut-conditionals. To investigate this further, additional experimental litters were generated by intercrossing *Grhl3* heterozygotes. PNP length was enlarged from as early as the 8–11 somite stage (at E8.5) in *Grhl3*^*-/-*^ embryos (Fig. 3B). This suggests that spinal neurulation becomes defective immediately after initiation of closure which typically occurs at the 5–7 somite stage. Failure of the initiation event (Closure 1) itself leads to craniorachischisis, which was not observed in *Grhl3*^*-/-*^ embryos, here or in previous studies (Ting et al., 2003a; Yu et al., 2006). Hence, the entirety of spinal closure, post-Closure 1, requires Grhl3 function.

We demonstrated a requirement for Grhl3 in the hindgut during later spinal neurulation, but *Grhl3* is not expressed in the hindgut at E8.5-9, when closure first fails (Fig. 3A). Therefore, Grhl3 function in another embryonic tissue must underlie this early-stage closure defect. In embryos with 8–11 somites, the surface ectoderm is the only tissue at the axial level of the ‘zippering point’ which expresses *Grhl3* (Fig. 3A), suggesting a vital role for Grhl3 function in this tissue for progression of closure. A putative role for the surface ectoderm in NTDs is consistent with the finding that initial contact of the neural fold tips in the spinal region is mediated by surface ectoderm cells at the border with the neural plate (Rolo et al., 2016). We analysed selected genes that function in epidermal differentiation in the caudal region of embryos at E9, shortly after onset of epidermal specification of the surface ectoderm. By qRT-PCR, *Trp63* (encoding TAp63)(Koster et al., 2007), a key transcriptional regulator of epidermal specification, and its target *Tfap2c* (Koster et al., 2007), were significantly down-regulated in *Grhl3* null embryos (Table S1), suggesting a possible delay in specification of the surface ectoderm. By E10.5, the basal keratin *Krt5* was also down-regulated in *Grhl3* null embryos (Table S2), although *Krt8* and *Krt18* (expressed in uncommitted surface ectoderm) were not significantly altered at E10.5.

Although *Grhl3* is only expressed in the surface ectoderm at the PNP closure site at E8.5, mRNA is also detected in the posterior part of the embryo corresponding to the node-streak border and caudo-lateral epiblast, where neuro-mesodermal progenitors (NMPs) are located (Henrique et al., 2015)(Fig. 3A). Later, at E9.5, mRNA is also detected in the neural plate of the PNP as well as in the hindgut endoderm even later, at E10-10.5 (Gustavsson et al., 2007) (Fig. 3A). Expression in the surface ectoderm and neural plate at E9.5 was confirmed using *Grhl3*^*cre/+*^ embryos, in which β-galactosidase is expressed from the *Grhl3* locus (Fig. S1A-C), consistent with previous studies of the *Grhl3*^*cre*^ allele (Camerer et al., 2010). Moreover, use of *Grhl3*^*cre*^ to recombine *R62R-YFP* for lineage tracing revealed expression in all surface ectoderm cells and in a mosaic pattern within the neuroepithelium and paraxial mesoderm (Fig. S1D-H)(Rolo et al., 2016), consistent with *Grhl3* expression in the NMP population. Hence, it remained possible that *Grhl3* expression in the neural plate and/or NMPs is also required for spinal closure.

**Fig. 4:**
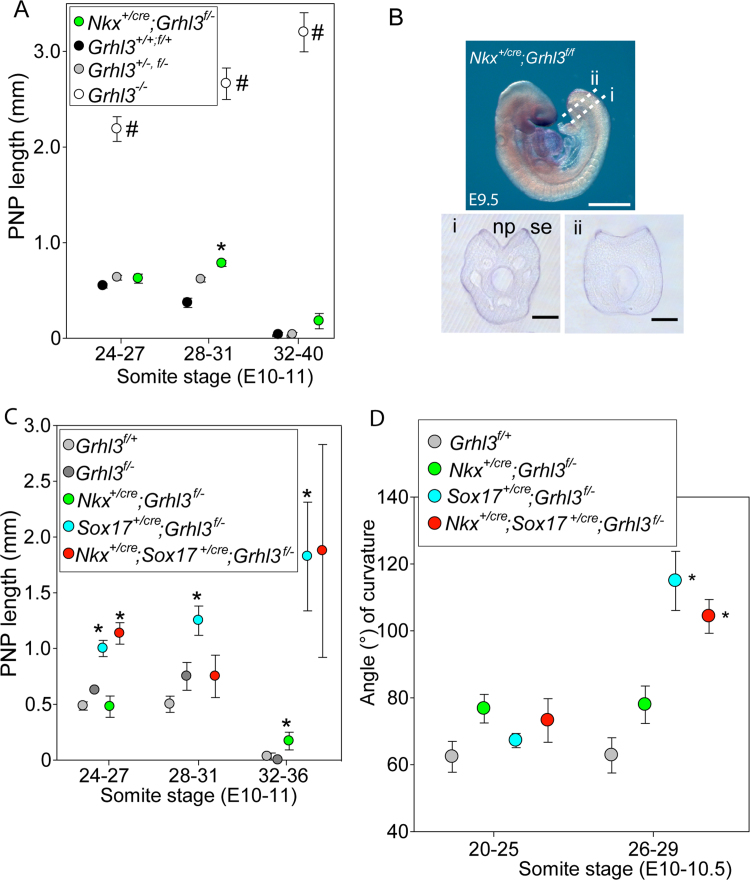
**Conditional deletion of*****Grhl3*****in the neural plate has little effect on PNP closure** (A) PNP closure in embryos in which *Grhl3* was conditionally deleted in the neural plate using *Nkx1-2*^*cre*^ appeared at most slightly delayed compared to wild-type embryos but with minimal effect compared with *Grhl3*^*-/-*^ (* significantly different from *Grhl3*^*+/+*^, p<0.001, ANOVA; # significantly different to all other genotypes, p<0.001, ANOVA). n = 57 *Grhl3*^*+/+, f/+*^, 49 *Grhl3*^*+/-, f/-*^, 27 *Grhl3*^*-/-*^, 28 *Nkx1-2*^*cre*^*; Grhl3*^*f/-*^ (4–24 embryos per group at each somite interval). (B) Sections through the caudal region of *Nkx1-2*^*+/cre*^*;Grhl3*^*f/-*^ embryos confirmed loss of *Grhl3* expression in the neural plate (white dashed lines in embryo indicate levels of sections in Bi-ii), compared with Fig. 3Aii’. (C) Conditional deletion of *Grhl3* in both neural folds and hindgut (*Nkx*^*+/cre*^*;Grhl3*^*f/-*^*;Sox17*^*+/cre*^; n = 16) results in a significant defect in PNP closure compared with *Grhl3*^*f/+*^ and *Grhl3*^*f/-*^ (n = 22). This appears largely attributable to the effect of conditional deletion in the hindgut endoderm as PNP lengths were similar to *Sox17*^*cre*^*;Grhl3*^*f/-*^ embryos (n = 15) generated in the same cross (* significantly different to *Grhl3*^*f/+*^, p<0.01, ANOVA; n = 3–10 per group at each somite stage). (D) The angle of curvature (measured as in Fig. 1) of the caudal region was significantly greater in *Nkx1-2*^*cre*^*; Sox17*^*cre*^*; Grhl3*^*f/-*^ and *Sox17*^*cre*^*; Grhl3*^*f/-*^ embryos than the other genotypes at the 26–29 somite stage (*p<0.001, ANOVA; n = 8 – 14 embryos per genotype).

Expression of early neural plate markers, *Sox2* and *Nkx1-*2, was not altered in the caudal region of *Grhl3* null embryos at E8.5-9.0 (Fig. 3C-F; Table S1), suggesting that initial specification of the neural plate and NMPs is not compromised in *Grhl3*^*-/-*^ embryos. To further examine potential functions of *Grhl3* in the neural plate, we generated conditional knockouts using *Nkx1-2*^*Cre*^ which drives recombination in the neuroepithelium of the PNP and NMPs, but leaves expression in the surface ectoderm intact (Fig. 4B) (Albors et al. http://dx.doi.org/10.1101/045872)(Rolo et al., 2016). *Nkx1-2* (previously *Sax1*) is expressed in the caudal lateral epiblast and in the open neural plate throughout neurulation stages (Rolo et al., 2016). Induction of *cre* prior to onset of neurulation and measurement of PNP lengths at E10.5 (in embryos from *Nkx1-2*^*cre/+*^*;Grhl3*^*+/-*^ x *Grhl3*^*f/-*^ matings) did not indicate a major defect of spinal neurulation among embryos lacking *Grhl3* expression in the neuroepithelium; the PNP length of *Nkx1-2*^*cre/+*^*;Grhl3*^*f/-*^ embryos never approached the size observed in *Grhl3*^*-/-*^ embryos (Fig. 4A). However, it was significantly larger than among wild-type embryos at E10.5 (28–31 somite stage). Part of this delay in PNP closure in *Nkx1-2*^*cre/+*^*;Grhl3*^*f/-*^ embryos can be attributed to heterozygosity for the null *Grhl3* allele as *Grhl3*^*+/-*^ and *Grhl3*^*f/-*^ embryos also showed a non-significant trend towards larger PNP length (Figs. 4A, 3B). However, the PNP was still open at E10.5 in some *Nkx1-2*^*Cre*^*;Grhl3*^*f/-*^ embryos at somite stages when all wild-type and heterozygous embryos had completed closure (2/9 compared with 0/13 *Grhl3*^*+/+*^ and ^f/+^ or 0/8 *Grhl3*^+/-^ embryos with 36 or more somites) suggesting that ablation of *Grhl3* expression in the neural plate and NMPs is sufficient to delay completion of spinal closure in a small proportion of embryos.

In order to further investigate the tissue-specific requirement for *Grhl3* expression, we performed additional experimental matings (*Nkx1-2*^*cre/+*^*;Grhl3*^*f/f*^ x *Sox17*^*cre/+*^*; Grhl3*^*+/-*^) to generate embryos in which the ‘floxed’ *Grhl3* allele was recombined in both hindgut endoderm and neural plate (*Nkx1-2*^*cre*^*; Sox17*^*cre*^*; Grhl3*^*f/-*^) but not surface ectoderm. In this cross, PNP closure was still incomplete at late E10.5 (32 or more somites) among 4/5 *Sox17-cre*, 4/6 *Nkx1-2-cre* and 2/3 *Sox17-cre; Nkx1-2-cre* embryos, compared with only 1/6 *cre*-negative *Grhl3*^*f/-*^ controls (Fig. 3C). As in the previous cross, the PNP length of embryos expressing *Sox17-cre* was significantly enlarged from the 24–27 somite stage onwards. The additional presence of *Nkx1-2-cre* did not lead to a further increase in mean PNP length (Fig. 3C). There was a significant increase in ventral curvature of the caudal region of *Sox17*^*Cre/+*^*;Grhl3*^*f/-*^ and *Sox17*^*Cre/+*^*;Nkx1-2*^*cre/+*^*;Grhl3*^*f/-*^ embryos compared with *Grhl3*^*f/+*^ controls (Fig. 3D). In contrast, ventral curvature of *Nkx2-1*^*cre/+*^*;Grhl3*^*f/-*^ embryos was comparable to controls (Fig. 3D). These findings support the hypothesis that it is the loss of *Grhl3* expression in the hindgut that is primarily responsible for excess curvature and failure of PNP closure.

Together, these data suggest that Grhl3 expression in the hindgut, and to a much lesser extent in the neuroepithelium, is required at the later stages of spinal closure at E10-10.5, whereas spinal neural tube closure first requires Grhl3 function in the surface ectoderm, from an early stage after the initial closure event at E8.5.

## 4 Discussion

Spinal neurulation appears to be highly dependent on Grhl3 with diminished expression, as in the *curly tail* (*ct/ct*) strain (Gustavsson et al., 2007), complete loss in *Grhl3*^*-/-*^; (Yu et al., 2006) or tissue-specific loss in the hindgut (this study) all causing failure of PNP closure, leading to spina bifida. During neural tube closure, *Grhl3* is expressed not only in the surface ectoderm, but also at additional sites (neuroepithelium, gut endoderm and node-streak border/caudo-lateral epiblast). Our findings demonstrate a requirement for *Grhl3* expression in at least two tissues, including the hindgut.

Previous studies suggested that NTDs in *curly tail* embryos result from defective hindgut cell proliferation that disrupts closure via a biomechanical effect of excess axial curvature on neural fold elevation and apposition (Van Straaten and Copp, 2001). The neuroepithelium of the PNP is tightly attached to the underlying notochord and hindgut by extracellular matrix (O'Shea, 1987), so that defective growth of the hindgut serves to biomechanically deform the overlying neural plate. Splinting of the caudal region to avoid development of body axis curvature rescues PNP closure in otherwise untreated *curly tail* embryos (Brook et al., 1991), further indicating the importance of this pathogenic mechanism for spinal NTDs. In line with this hypothesis, rescue of PNP closure by inositol (Cogram et al., 2004), nucleotides (Leung et al., 2013) or *Grhl3*-BAC transgenesis (Gustavsson et al., 2008) is associated with stimulation of proliferation in the hindgut. Moreover, polymorphic variants of lamin B1 that differentially affect NTD frequency in *curly tail* embryos show concordant differences in cell proliferation (de Castro et al., 2012).

In the present study, we obtained strong supporting evidence for *Grhl3* involvement in the hypothesised mechanism of *curly tail* spinal NTDs, in particular from the finding of low spinal NTDs in gut-specific conditional *Sox17*^*cre*^*; Grhl3*^*f/-*^ mutants. This observation provides an independent demonstration of the requirement for *Grhl3* expression in the hindgut, during completion of PNP closure. Additional evidence includes the relatively late stage at which PNP closure first becomes abnormal in both *curly tail* and *Sox17*^*cre*^*; Grhl3*^*f/-*^ mutant embryos (E10), which correlates with the stage of onset of *Grhl3* expression in the hindgut during wild-type development.

Grhl3 is presumably required, therefore, to promote a normal rate of cell proliferation in the embryonic hindgut, at least at neurulation stages. However, studies in other systems implicate Grhl3 as a negative influence on cell proliferation. For example, in developing skin, *Grhl3* mutations cause hyper-proliferation (Ting et al., 2005), while in normal tongue papillae, loss of Grhl3 results in enhanced cell proliferation (Adhikari et al., 2017). Hence, the specific requirement for Grhl3 in hindgut cell proliferation remains to be determined.

In addition to the relatively late requirement for *Grhl3* expression in the embryonic hindgut, stage-dependent analysis shows there is also an earlier requirement for Grhl3 in spinal neurulation. Null embryos develop severe NTDs owing to early onset of abnormal PNP closure, from E8.5, more than a day before this is observed in endoderm-specific mutants. While closure initiation (Closure 1) occurs successfully, the whole of subsequent spinal closure fails in *Grhl3*^*-/-*^ embryos, generating extensive spina bifida. Several lines of evidence suggest a vital role of Grhl3 in the surface ectoderm at this early stage (8–11 somites) when closure starts to be delayed. First, Grhl3 expression can only be detected in the surface ectoderm at this stage. Expression in the neuroepithelium does not begin until 12 or more hours later, and hindgut expression has an even later onset. Second, conditional inactivation of Grhl3 specifically in the neuroepithelium using *Nkx1-2*^*cre*^ is not detrimental to neural tube closure, arguing against a key role for Grhl3 in the neuroepithelium. Third, genetic markers of surface ectoderm establishment and differentiation are reduced in expression in the caudal region of Grhl3^-/-^ embryos at the stage when neural tube closure defects first become apparent. Hence, Grhl3 expression in the surface ectoderm appears essential for the early stages of spinal neural tube closure in the mouse embryo.

A priority for future work is to conduct a conditional gene targeting approach to specifically eliminate Grhl3 function from the early surface ectoderm, in order to directly test its hypothesised role in early spinal neurulation. This approach was not possible in the present study, owing to the lack of a suitable cre-driver for early surface ectoderm. While Keratin14-cre is frequently used to eliminate gene function in the epidermis (Jonkers et al., 2001), we found that significant cre-mediated recombination occurs only following completion of spinal neurulation (data not shown), making K14-cre unsuitable for this study. The only other commonly used cre-driver for the surface ectoderm is *Grhl3*^*cre*^ itself, which is clearly precluded from use in the present study. Production of novel reagents to target the early surface ectoderm are required to enable its developmental roles to be fully evaluated.

In conclusion, *Grhl3* null embryos are subject to two successive closure-preventing insults: an initial surface ectoderm-mediated defect that hampers closure during early spinal neurulation, and a subsequent inhibitory influence imposed by caudal curvature resulting from lack of *Grhl3* expression in the hindgut. The latter causes low spina bifida in homozygotes for the *Grhl3*^*ct*^ allele, and reinforces the earlier closure delay in *Grhl3*^*-/-*^ null embryos. In terms of clinical relevance, this study provides proof-of-principle for a multi-hit model of birth defects resulting from a single key gene with distinct, critical functions in sequentially developing embryonic tissues.
