# Supplementary material for: Neural tube closure depends on expression of Grainyhead-like 3 in multiple tissues
Source: Dev Biol. 2018 Mar 15;435(2):130–7. doi: 10.1016/j.ydbio.2018.01.016 (PMC5854268; doi:10.1016/j.ydbio.2018.01.016)
Supplement: Supplementary file 1 — Supplementary material [file mmc1.docx]

**Supplementary Figures**

**
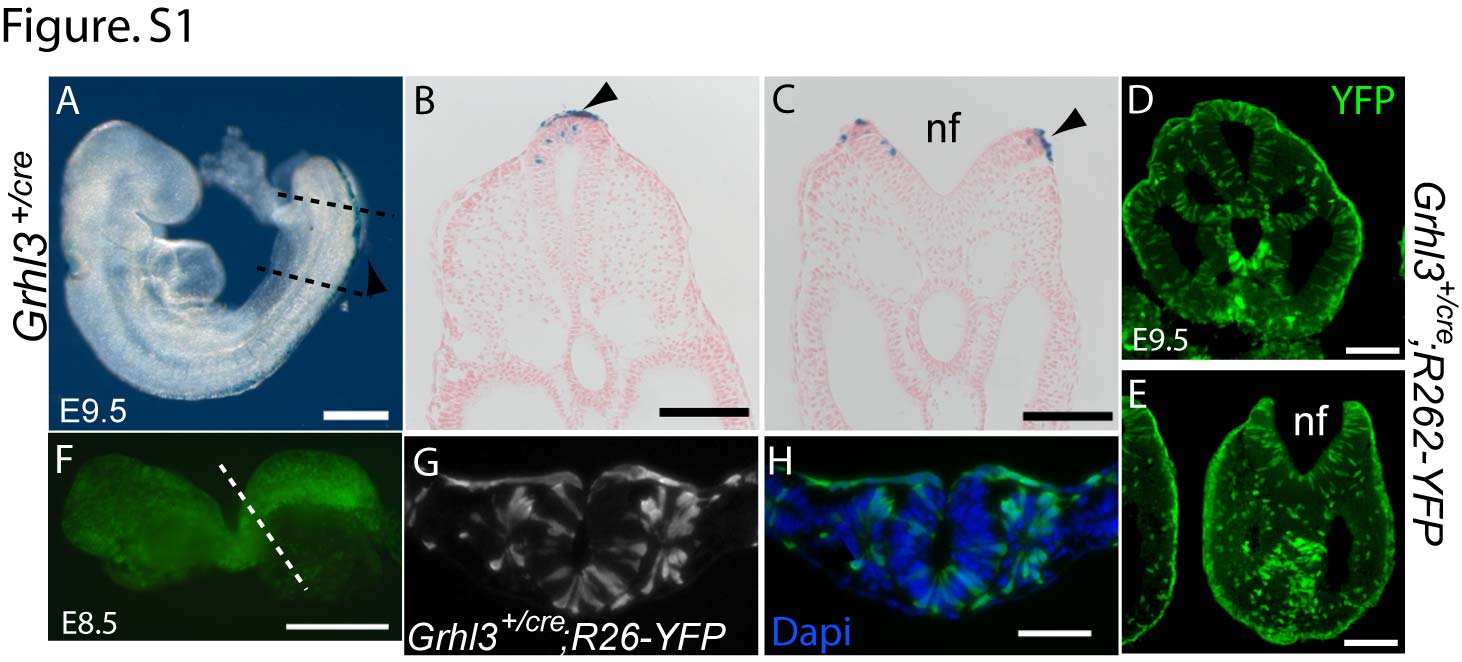
**

**Figure S1. Expression of *lacZ* and activity of *Cre* recombinase expressed from the *Grhl3* locus.** (A-C) X-gal staining shows expression of *lacZ* from the *Grhl3* locus in *Grhl3^cre/+^* embryos at E9.5 (16 somite stage), with β-galactosidase staining in the surface ectoderm and scattered positive cells in the open neural plate (C) and closed neural tube (B). Dotted lines (A) show level of sections in B and C; black arrow shows rostral extent of the posterior neuropore. (D-H) YFP-positive cells in *Grhl3^cre/+^;R62R-YFP* embryos, representing cells or their descendants that expressed cre-recombinase from the *Grhl3* locus. Lineage-traced YFP-positive cells are observed in the surface ectoderm (all cells positive), with mosaic expression also in the neuroepithelium and mesoderm at E9.5 (D-E) and E8.5 (F-H). This is consistent with prior expression of *Grhl3* in the region of the node-streak border where neuromesodermal progenitors arise.


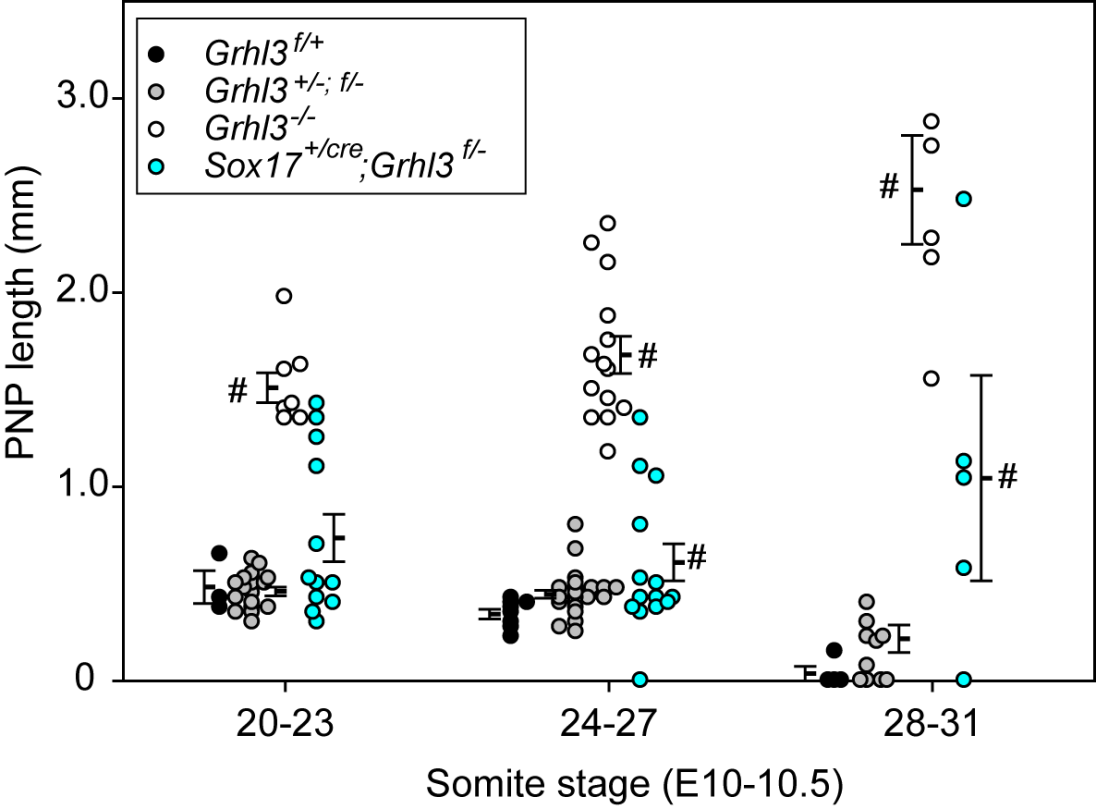


**Figure S2. Posterior neuropore (PNP) length at E10-10.5 among offspring of *Grhl3^f/-^* intercrossed with *Grhl3^f/-^*; *Sox17^+/cre^*.** The graph shows data for individual embryos. Mean ± SEM is indicated for each group (as shown in Fig. 3D); # indicates significant difference compared to all other genotypes (p<0.001; ANOVA).

| Genotype | *No. samples* | *Grhl3* | *Trp63* (p63) | *Tfap2c* (AP2γ) | *Sox2* | *Nkx1-2* |
| --- | --- | --- | --- | --- | --- | --- |
| *Grhl3^f/f^* ^or^ *^f/+^* | 5-7 | 0.95 ± 0.05 | 1.00± 0.04 | 0.95 ± 0.06 | 1.1 ± 0.1 | 1.1 ± 0.1 |
| *Grhl3^-/-^* | 4-5 | **0.05 ± 0.02**** | **0.66± 0.06**** | **0.50 ± 0.01**** | 1.1 ± 0.4 | 1.1 ± 0.4 |

**Table S1. Expression of epithelial/epidermal markers in the caudal region of *Grhl3* null embryos at E9.0-9.5.** mRNA abundance was determined by qRT-PCR with data normalised to the control (*Grhl3^f/f^* or *Grhl3^ct/ct^*) within each group. Values represent mean ± SEM. **significant difference from control group (p<0.005; ANOVA and pairwise comparison).

| Genotype | *No. samples* | *Grhl3* | *Krt8* | *Krt18* | *Krt5* | *Tgm1* | *Cdh1* |
| --- | --- | --- | --- | --- | --- | --- | --- |
| *Grhl3^f/f^* ^or^ *^f/+^* | 3-7 | 1.02±0.07 | 0.89 ± 0.15 | 1.12 ± 0.12 | 1.02 ± 0.15 | 0.93±0.08 | 1.00 ± 0.0 |
| *Grhl3^-/-^* | 4-8 | **0.02±0.02**** | 0.66 ± 0.07 | 0.97 ± 0.16 | **0.56 ± 0.12*** | **0.53±0.06**** | **0.70 ± 0.05*** |

**Table S2. Expression of known targets of *Grhl3* in epithelia or epidermis in the caudal region of *Grhl3* null embryos at E10.5.** Genes encode keratins (*Krt5*, *8*, *18*), loricrin (*Lor*), transglutaminase 1 (*Tgm1*) and E-cadherin (*Cdh1*). mRNA abundance was determined by qRT-PCR with data normalised to the control (*Grhl3^f/f^* ) within each group. Values represent mean ± SEM. Significant difference from control (*Grhl3^f/f^*) within group (**p<0.005, *p<0.05; ANOVA). ND: not determined.
